# Effect of anthropometrical measurements on portal vein and hepatosplenic span

**DOI:** 10.12669/pjms.294.3617

**Published:** 2013

**Authors:** Tanya Raza Siddiqui, Nuzhat Hassan, Pashmina Gul

**Affiliations:** 1 Dr. Tanya Raza Siddiqui, MBBS, M. Phil Candidate, Senior Lecturer, Department of Anatomy, Ziauddin Hospital, Karachi, Pakistan.; 2 Dr. Nuzhat Hassan, M.phil- Anatomy, Head of Department of Anatomy, Ziauddin Hospital, Karachi, Pakistan.; 3 Dr. Pashmina Gul, FCPS, Assistant Professor, Department of Radiology, Ziauddin Hospital, Karachi, Pakistan.

**Keywords:** Portal vein, Liver, Spleen, Anthropometrical measurements, Doppler ultrasonography

## SUMMARY

Deaths from liver disease have doubled over the last fifteen years. Chronic liver disease and cirrhosis are still the twelfth leading cause of death in the United States. Till date no comprehensive data has been documented in our part of the world which can help a clinician in diagnosing organomegaly. Improved hardware and knowing the exact dimensions of an organ with respect to its anthropometrical measurements can be of great importance in a radiological clinical routine. This article review was the result of recent clinical studies relating to portal vein, liver and spleen normograms in different populations which can show a comparison in accordance with their anthropometrical factors.

***Sources of Data/Study Selection: ***Data from survey reports, cross-sectional and prospective studies published between the years 2003-2012 on the topic were included. Data searches included both human and cadaveric studies.

***Data Extraction: ***The data was extracted from online resources of statistic reports, Pub med, THE MEDLINE, Google, Medical and Radiological journals.

***Conclusion: ***Sonographical analysis of the effect of anthropometrical measurements on the dimensions of portal vein, liver and spleen can be important markers for evaluation, diagnoses and assessment of portal hypertension, organomegaly and liver transplantation.

## OVERVIEW

***Effect of age, gender and BMI on the diameter and velocity of portal vein: ***All the nutrient rich blood from the GIT flows into the portal vein which determines the anatomical division of the hepatic lobes.^1^^,^^2^ The length of the portal vein is of surgical importance which is often cut down to link with the other vessels in interventional procedures like liver transplantation, trans hepatic portal vein embolization and pancreatectomy.^3^^-^^5^ It is an established fact that the normal hepatopetal flow in portal vein is affected in diseased individuals but it can also vary considerably in normal individuals making it a strong predictor for future portal vein diseases.^2^^,^^3^^,^^6^ Several studies have been performed to establish the normal upper limits of the portal vein diameter (PVD) but these values vary according to the sonographer, mode of technique and the population being studied upon.^3^^,^^4^^,^^7^^,^^8^ Literature review has shown that the most primitive diameter of portal vein was established as 6.3 ± 2.3mm.^6^

A cross sectional gray scale ultrasound assessment of portal vein in Ethiopian population was done, age of the subjects varied from 5 to 85 years. The mean diameter of portal vein was calculated as 10.0+1.8cm.^9^ The results concluded that gender did not have any effect on the diameter of portal vein but with increasing age the diameter also increased.^9^ Subsequently, Anakwue et al also reported the same results in a Nigerian population establishing the mean portal vein diameter as 11.45+1.45mm and also concluding that the diameter varies with age but not with gender.^6^ In contrast to these researches a Doppler ultrasonic study which is a more advanced and accurate imaging technique was conducted in Iran on 37 healthy subjects. The age varied from 20-40 years and the mean portal vein diameter was calculated as 9.4 ± 1.7 mm.^10^ The mean portal vein velocity (PVV) was established as 27.317±13.139.^10^ Though the sample size of the study was small yet it was suggested that gender, phases of respiration and measuring techniques also had a significant effect on portal vein as backed up by other researches.^4^^,^^7^ Despite of this published data a color doppler ultrasound of the main portal vein was conducted in Malaysian population concluding that no significant proof was found in the diameter and velocity between gender, age and change in position.^11^

A recent study conducted in India by Ravi Shankar et al on a large sample size concluded that in males the portal vein diameter did not vary with age but height had a positive correlation with PVD.^7^ In the same study, PVD in the female subjects was found to have no correlation with age or height.^7^ However, one of the limitation of this study was that only the diameter of portal vein was assessed and not the portal flow which is also an important marker for the diagnosis of hepatobilliary diseases.^7^

Anatomical divisions of portal vein have also been observed in a cadaveric study in Srilanka. The mean diameter of PV as 8.96+1.26mm and the length as 8.28+1.26cm were established. When compared with the other values published, it was concluded that this population had longer lengths and shorter diameters. This may be because of difference in ethenicity.^3^

Serife et al compared portal vein velocity in patients with non alcoholic fatty liver disease (NAFLD) and healthy control group suggesting that patients with NAFLD had low portal vein velocity reinforcing the fact that both portal vein size and velocity are diagnostic tools in measuring portal hypertension and liver cirrhosis.^12^

Therefore, anatomical variations of portal vein (PV) according to anthropometrical data exists in each population which lacks statistical documentation.^6^^,^^7^^,^^9^^,^^10^

***Effect of anthropometrical measurements on liver span: ***Ailments comprising from inflammatory conditions to malignant scenarios can affect the liver span and in order to treat these disorders knowing the normograms of liver is imperative,^2^^,^^8^^,^^13^ specially, while assessing healthy donors for liver transplant.^5^^,^^14^^,^^15^

Recently, a pilot study was conducted on a Rajisthani population to know the normograms of liver in correlation with age and gender. Both the parameters were found to have a positive influence on the hepatosplenic span.^8^ However, BMI was not taken into consideration which could have guided a clinician for better diagnosis of organomegaly. Similarly, to evaluate the effect of anthropometrical measurements on the span of liver in adult Jordanian population Tarawneh ES et al suggested that the best predictor of female liver span is body surface area and in males it is the height measurement.^16^ A novel research to estimate the liver volume in north Indian population was done establishing that body height and age had the best correlation with liver volume than other body indices.^15^ Also a maximum peak of volume was observed in early twenties and later on a decline was observed with age advancement.^15^ The limitation reported in this study was that liver volume was not compared with the real size of the liver.^15^

The liver size increases from infancy to adult hood reaching its maximum size by the age of 15 years.^14^^,^^17^ However influences of age, gender, dietary status, examination technique and fatty infiltration even in a healthy population cannot be ignored.^14^^,^^17^^,^^18^ This literature review was also supported by Tetsuya et al who have worked on liver grafting in transplantation procedures.^14^ Another study conducted by Benjamin et al also concluded that in the South Eastern Nigerian population males had a larger liver size than females and that with age the size increases where as at the age of fifty progressive decrease in the liver span was observed.^19^

In contrast to these researches, Wolfgang et al concluded in a massive sonographical survey that out of all the quantitative variables like height, age and BMI only the body height and BMI had a clinical significance on the size of liver.^20^ In qualitative variables gender was also found to have no affect on the liver span. Intraobserver variability was one of the limitation of this study as data was collected by eight radiologists.^20^ Other studies have also cited that in slender individuals the liver is oriented longitudinally while in heavy subjects it is transverse.^19^ Rosemeri et al concluded that in the Caucasian population there was no significant difference in the liver size of individuals whose BMI is less than 25kg/m^2^ when measured either by ultrasonography or physical examination. Only individuals whose BMI was more than 25kg/m^2 ^the correlation between these two techniques was significant.^18^

We can confidently summarize that till date no uniform concensus has been agreed upon the normal liver span and each population should have its own reference range. Hence, effect of anthropometrical measurements and technique of measurement should all be taken into account when evaluating a patient.

***Factors affecting spleen span: ***Spleen is a functionally diverse organ. The normal spleen is usually not palpable but it can sometimes be palpated in adolescents and individuals with a slender build. Determination of spleen size is important because in diseased conditions specifically leukemia; it is two to three times enlarged before being clinically palpable.^13^^,^^21^

Asghar A et al did a significant prospective study on the splenic volume estimation concluding that the splenic volume had a linear correlation with the body height and this factor can be further investigated in individuals suspected of splenomegaly to avoid false positive diagnoses of splenomegaly.^22^ The same results were found by Speilmann et al suggesting that spleen size had a positive correlation with height in both male and female athletes.^23^ Mittal R et al conducted a pilot study on estimation of spleen size on Rajisthani population concluding that the males had a greater spleen length as compared to females and with age the size also increased.^8^ Ehimwenma et al also conducted a similar survey in Nigeria showing a statistical difference in splenic dimensions of males and females however no statistical correlation was found with age in either gender.^24^

In a recent study conducted by Mustapha et al in which spleen size was correlated with age, gender, height, weight and BMI only gender correlation came out to be positive and that mean splenic volume in African adults was lesser than that of published in western population.^25^ However, splenic volume measurements were correlated with anthropometrical measurements recently in Japan by Harris et al and out of all the anthropometric factors only body weight was found to be statistically positive.^26^

Hence, measurement of spleen size in correlation with anthropometric measurements is a useful predictive indicator which can be used in daily radiological clinics.

***Techniques for determining the size of portal vein, liver and spleen: ***To establish the precise measurement of any organ by an accurate and concrete modality is of utmost importance for diagnosis and guidance of the treatment procedures. Various techniques have evolved over a period of time from simple bedside examination to more sophisticated and invasive procedures but they have been debated over for various reasons as discussed below.

Percussion and palpation are the standard bedside techniques to document liver and spleen size, but not reliable and accurate to detect the small increase in size.^27^ Clinical studies have already proved that radiography and radionuclide studies expose the patient to gamma radiations which have harmful effects.^27^ Doppler ultrasound uses high frequency non ionizing waves which have virtually no side effects. It is cost effective, no pain or discomfort is experienced by the patient and gives fast and homogenous results.^28^^,^^29^

In a recent pictorial essay of portal system diseases by a multidetector CT scan Ozbulbul et al have reinforced the importance of portal venous system which will lead to a definitive diagnosis.^30^ Karnam et al have also cited ultrasonography as a well suited technique for imaging of hepatobilliary tract.^31^ In another recent research the role of ultrasound was reviewed in portal hypertension concluding that it should be the investigation of choice.^32^ However, In a recent Doppler assessment of liver disease O’ Donohue J et al concluded that the measurements of PVD, PVV and hepatic arterial resistance index was the same in cirrhotic and healthy individuals. Reason was attributed to interobserver variability.^33^ Similarly, Shetri K et al concluded that in grading of cirrhosis sonographic dimensions of PVD and PVV are not reliable indicators.^28^ Benter T et al in a pictorial essay of sonography of spleen concluded that though computed tomography and magnetic resonance imaging are the latest techniques available but sonography still plays a pivotal role in emergency diagnosis of splenic rupture and hemorrhage.^34^

By assessing the hepatosplenic span by an accurate imaging procedure both radiologists and clinicians can improve the accuracy of assessment of hepatobilliary diseases in all age groups.

## CONCLUSION

Keeping in mind the alarming increase of hepatitis B and C in our country till date, no comprehensive data on the normograms of portal vein and hepatosplenic span with respect to anthropometrical measurements has been documented. This article has summarized the effects of age, gender and body mass index on the present values of portal vein diameter, velocity and liver spleen span in different ethnic backgrounds. Hopefully, future researches can lead to evolution of clinical strategies in radiological setups which can be significant in the diagnosis of portal hypertension and for selection of subjects for liver transplant. Furthermore, aspects of interobserver variability and intraobserver variability in the field of ultrasonography are also important to note in order to eliminate the false positive diagnosis of organomegaly.

## Authors Contribution:

Dr. Tanya Raza Siddiqui: Concept, design of the study and writing the manuscript

Dr. Nuzhat Hassan: Critical analysis of the manuscript.

Dr. Pashmina Gul: Clinical analysis and final approval of the manuscript.
